# Interactions between oestrogen and 1α,25(OH)_2_-vitamin D_3_ signalling and their roles in spermatogenesis and spermatozoa functions

**DOI:** 10.1186/s12610-017-0053-z

**Published:** 2017-05-08

**Authors:** Ana Paula Zanatta, Vanessa Brouard, Camille Gautier, Renata Goncalves, Hélène Bouraïma-Lelong, Fátima Regina Mena Barreto Silva, Christelle Delalande

**Affiliations:** 1 INRA, OeReCa, Normandie University, UNICAEN, 14000 Caen, France; 2 Biochemistry Department, Laboratory of Hormones & Signal Transduction, UFSC, Florianópolis, Brazil; 3 Laboratoire Œstrogènes, Reproduction, Cancer (OeReCa), EA 2608 USC INRA1377, Université de Caen Normandie, Esplanade de la Paix, CS 14032, 14032 CAEN cedex 5, France

**Keywords:** Œstrogènes, 1,25(OH)_2_-vitamine D_3_ (1,25-D_3_), Spermatogenèse, Spermatozoïde, Récepteurs aux stéroïdes, Oestrogen, 1α,25(OH)_2_-vitamin D_3_ (1,25-D_3_), Spermatogenesis, Spermatozoa, Steroid receptors

## Abstract

Oestrogens and 1α,25(OH)_2_-vitamin D_3_ (1,25-D_3_) are steroids that can provide effects by binding to their receptors localised in the cytoplasm and in the nucleus or the plasma membrane respectively inducing genomic and non-genomic effects. As confirmed notably by invalidation of the genes, coding for their receptors as tested with mice with in vivo and in vitro treatments, oestrogens and 1,25-D_3_ are regulators of spermatogenesis*.* Moreover, some functions of ejaculated spermatozoa as viability, DNA integrity, motility, capacitation, acrosome reaction and fertilizing ability are targets for these hormones. The studies conducted on their mechanisms of action, even though not completely elicited, have allowed the demonstration of putative interactions between their signalling pathways that are worth examining more closely. The present review focuses on the elements regulated by oestrogens and 1,25-D_3_ in the testis and spermatozoa as well as the interactions between the signalling pathways of both hormones.

## Résumé

L’œstradiol et la 1α,25(OH)_2_-vitamin D_3_ (1,25-D_3_ ou calcitriol) sont respectivement la forme la plus active des œstrogènes et la forme hormonalement active de la vitamine D. Ces stéroïdes peuvent exercer leurs effets biologiques après fixation à des récepteurs localisés dans le cytoplasme et le noyau (récepteurs dit nucléaires) ou par fixation à des récepteurs localisés à la membrane plasmique (récepteurs membranaires) à l’origine d’effets appelés génomiques et non génomiques respectivement. Bien que les œstrogènes aient longtemps été considérés comme uniquement des hormones féminines, de nombreux travaux ont permis de montrer leur importance dans le bon déroulement de la spermatogenèse et la qualité des gamètes. De même, la 1,25-D_3_ est capable de réguler les fonctions testiculaires suggérant son importance dans la fertilité. Les études réalisées sur leurs mécanismes d’action, bien qu’ils ne soient pas complètement élucidés, ont permis de mettre en évidence des interactions entre les voies de signalisation de ces deux hormones. Cette revue est centrée sur les évènements régulés par les œstrogènes et la 1,25-D_3_ dans les testicules et les spermatozoïdes et les interactions entre leurs voies de signalisation.

## Background

Spermatogenesis is a complex biological process under the control of interplay of autocrine, paracrine and endocrine factors. In addition to gonadotropins and androgens, it is now well known that oestrogens could play a significant role in the regulation of the events of mammalian spermatogenesis but the mechanisms of oestrogens effects are not well established (for review [1]). Moreover, some data showed 1α,25(OH)_2_-vitamin D_3_ (1,25-D_3_) implications in some events of spermatogenesis where 1,25-D_3_ could, as well as steroids, act at genomic (gene expression regulation) and at non-genomic levels (initiated at the plasma membrane) (for review [2]). To this aim, their classical receptors classified as nuclear receptors could also be localised in the cytoplasm or in the plasmatic membrane. So, some interactions between the signalling pathways of oestrogens and 1,25-D_3_ could be identified; this suggests the existence of common roles in spermatogenesis and spermatozoa functions. This review is focused on:

(i) oestrogen and 1,25-D_3_ receptors, their localization and signalling pathways in testicular cells and spermatozoa.
(ii) oestrogen and 1,25-D_3_ effects and their roles in the events of spermatogenesis and maturation of spermatozoa.
(iii) the signalling pathways interactions of both hormones.

Considering this data (mainly tested on both rodent and human species), it does ultimately provide insight into unresolved issues and future investigations.

## Oestrogen

### Oestrogen receptors in testis and spermatozoa

**Fig. 1:**
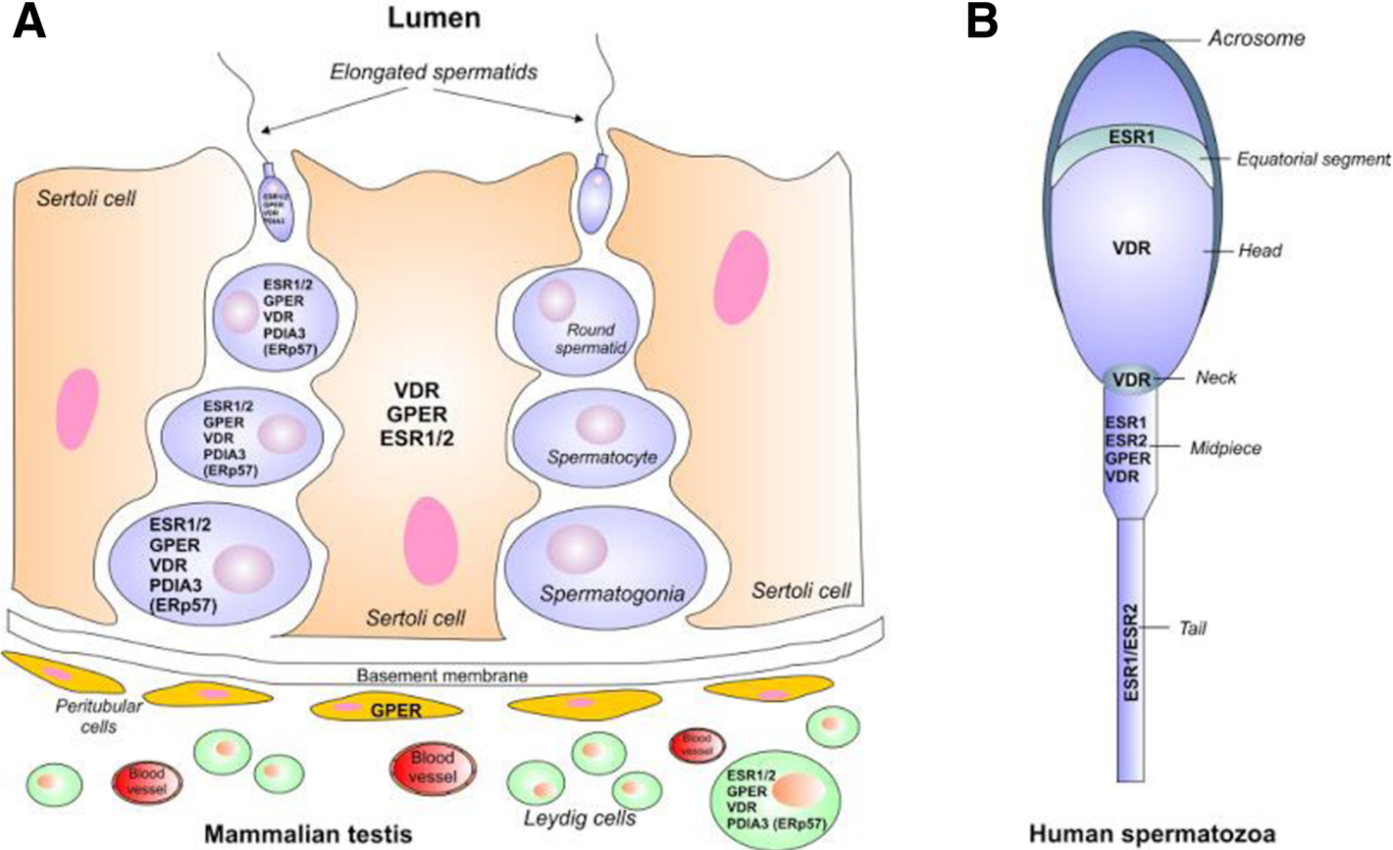
Oestrogen and vitamin D receptors in mammalian testicular cells and human spermatozoa. **a** Detection of ESR1, ESR2, GPER and VDR in the different testicular cell types. **b** Localization of ESR1, ESR2, GPER and VDR in human spermatozoa

Classically, oestrogens produce genomic effects after their binding to the nuclear receptors ERalpha or ESR1 and ERbeta or ESR2. However, they can also come and attach to receptors localised on the plasma membrane and activate some signalling pathways (for review [3]). In mammalian, both forms ESR1 and ESR2 are retrieved in somatic and germ cells in testis and on spermatozoa. Although some data values contradict each other the full length receptors ESR1 and ESR2 can be both found in Leydig, Sertoli and germ cells from spermatogonia to spermatids in human and rodents species (Fig. 1a; for review [1, 4, 5]). Recently, *in situ* hybridisation and immunohistochemistry experiments on human testis confirmed ESR1 and ESR2 expression in germ cells, ESR1 expression in interstitial cells and ESR2 expression in Sertoli cells [6]. In addition, some variants have been found. On human beings, six *ESR2* mRNA variants, in addition to the wild type receptor, are present in testis and their different localisation suggests that they undertake different roles in spermatogenesis [7]. In addition to the wild type *ESR1* mRNA, human germ cells express some transcripts deleted from exon 1 whereas the human spermatozoa exclusively contain the truncated form [8]. Consistently with these results, the presence of a shorter isoform of ESR1 of 46 kDa could be observed on human spermatozoa [8] instead of the expected size receptor of 66/67 kDa detected by Aquila et al. [9] and Rago et al. [10]. Later on, these two forms of 66 and 45 kDa were both detected by Solakidi et al. [11] (Fig. 1b). Moreover, a 29 kDa protein was identified by western blotting in human sperm membranes [12]. Saunders and collaborators described the expression of two proteins ESR2 in human adult testis as such: ERβ1 (wild type) and a variant isoform of ERβ(hERβcx/2) formed by alternative splicing [13]. The two proteins ERβ1 and ERβ2 were retrieved in germ cells, which are in keeping with the two proteins of 60 kDa and 50 kDa observed in human immature germ cells [8] and ejaculated immature spermatozoa [10]. Whereas no ESR2 protein was detected in human spermatozoa in the earlier phases [8], a 64 kDa protein was finally detected later on [11, 14]. ESR1 and ESR2 were observed on the tail or/and in the mid-piece with an additional localization for ESR1 to the equatorial segment [11, 14] (Fig. 1b). Although the corresponding proteins could not have been observed until now, nevertheless, we have also identified in rat testis some mRNA variants of ESR1 and ESR2. Concerning ESR1, we have shown the presence of the full-length form and of one isoform with exon 4 deleted. For ESR2, besides the wild type, three isoforms were observed: one with exon 3 deleted and another with an insertion of 54 nucleotides, and the last one with both modifications [15]. In addition to ESR1 and ESR2, a transmembrane receptor coupled to a protein G: GPER (GPR30: G protein coupled receptor 30) is able to bind to estradiol and to mediate its effects through a non-genomic pathway (for review [16]). This protein can be found in the mouse spermatogonial cell line GC-1 and in the mouse spermatocyte-derived cell line GC-2 [17, 18] but also in the rat primary germ cells PS (pachytene spermatocyte) and RS (round spermatid) [4, 5], and in immature rat Sertoli cells [19]. In human testis, GPER was also described in Sertoli cells and germ cells (spermatogonia and spermatocytes) and exclusively overexpressed in seminomas, the most frequent testicular germ cell cancer [20]. But, then, GPER was predominantly described in peritubular cells [21] (Fig. 1a). These results are in part different from data on *GPER* gene expression, which was observed weakly in Sertoli cells and higher in interstitial cells [6]. The GPER protein of 42 KDa was retrieved on human spermatozoa, in the mid-piece (Fig. 1b) but its localization seems to be species dependent [22]. Therefore, the three forms of oestrogen receptors are expressed by testicular cells and spermatozoa.

### Roles, effects and signalling pathways of oestrogens in testis and spermatozoa

#### KO and gene overexpression: mice models and men mutations

Some evidence on the role of oestrogens in male fertility came from the data obtained in the mouse knockout (KO) model. Especially, while male mice deficient in aromatase (*Cyp19*KO) were initially fertile, they developed progressive infertility with time. Disruptions of spermatogenesis were observed between 4.5 months and 1 year, despite no decreases in gonadotropins or androgens. Spermatogenesis was primarily stopped at early spermiogenic stages as characterized by an increase in apoptosis and the appearance of multinucleated cells, leading to a significant reduction in round and elongated spermatids without reduction changes in Sertoli cells and earlier germ cells. In addition, the presence of Leydig cells hyperplasia/hypertrophy could be clearly appreciated; presumably as a consequence of increased circulating luteinizing hormone [23]. The ERαKO mice were infertile [24] as the ERαβKO mice [25, 26]. Although spermatogenesis was normal up to 10 weeks, mice presented disrupted spermatogenesis and later degeneration of seminiferous tubules afterwards; this was due to the inability to reabsorb luminal fluids [24]. The same phenotype was revealed during the experiment on mice which were deleted from exon 3 of the *Esr1* gene lacking the DNA binding domain and null for ERα (Ex3αERKO mice) [27]. Joseph and collaborators have observed that sperm recovered from the epididymis of ERαKO mice revealed abnormal coiled flagellum and increased the incidence rate of spontaneous acrosome reactions [28, 29]. To determine the importance of non-classical action of oestrogen receptor, a mouse model expressing exclusively an ERα mutant (2 aa mutation in the DNA binding domain) was produced [30]. It allowed us to demonstrate that non-ERE-dependent oestrogen pathways are sufficient to rescue the defective spermatogenesis observed in ERKO mice and play a prominent role in ERα action in the testis, including pathways that regulate water resorption and androgen biosynthesis. Moreover, oestrogen non responsive ERalpha knock-in (ENERKI) mice model (mutation in the ligand-binding domain) permitted to enlighten that oestrogen dependent and independent oestrogen receptor alpha signalling separately regulate male fertility, with an essential role for oestrogen independent ER signalling to concentrate epididymal sperm via regulation of efferent ductule fluid reabsorption and a necessity of oestrogen-dependent ER signalling for germ cell viability [31]. The first model of ERβKO mice was fertile [32] whereas the ERβKO mice developed by Antal later in which exon 3 was deleted through Cre/LoxP-mediated excision and which was devoid of any transcript downstream exon 3 were sterile. However, these mice presented no histopathological abnormalities [33]. Otto and collaborators suggest that GPR30 does not mediate oestrogenic responses in reproductive organs in mice as GPR30KO mice presented no difference in their litters as compared to wild type mice [34], however there was no description of the spermatogenic process.

Over-expressions of the *Cyp19* gene encoding for aromatase enzyme were conducted and brought to light that transgenic male mice expressing human P450 aromatase (AROM+) were infertile (for review [35]).

Only eight cases of invalidation of aromatase have been reported in human beings (for review [36]),[37]. Among them, some patients presented an impaired reproductive function characterised by a decrease of motility and the number of spermatozoa (for review [36]). Only one case of congenital deficiency in oestrogens was described in human beings, due to a resistance to oestrogens consecutive to a punctual mutation of *ERα* gene, which revealed a higher sperm density than normal but a reduction in viability [38]. In human beings, polymorphisms of oestrogen related genes would mainly regulate sperm concentration and motility but not sperm morphology [39]. Guardacci and collaborators have observed an inverse correlation between higher TA, repeated in the promoter region of the *ERα* gene and the total sperm number in a group of infertile men, suggesting a possible negative influence on human spermatogenesis [40]. The long TA repeats would enhance oestrogen action [41]. The significance of the *ERα* gene in spermatogenesis and semen quality was supported by the data of Lazaros and collaborators who observed *ERα* polymorphisms associated to sperm motility and concentration [42]. Whereas the single nucleotide polymorphisms (SNPs) and *ERβ* gene mutations don’t seem to be a common cause of spermatogenesis failure in Indian men [43], Aschim and collaborators [44] concluded that some SNPs in *ERβ* could modulate human spermatogenesis. They observed a frequency of the heterozygous RsaI AG-genotype three times higher in infertile men compared to controls [44]. However, no significant associations were found between *ERβ* (1082G → A and 1730A → G) polymorphisms and sperm concentration or motility [42].

#### In vivo treatments

**Fig. 2:**
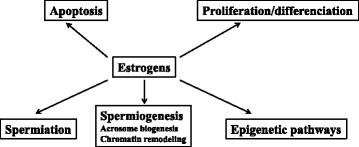
Oestrogen roles in mammalian testis

Studies conducted in rodents and primates have shown that spermatogenesis is partly under oestrogens control at different levels. In the immature bank vole, exposure to a low dose of estradiol induced acceleration of the onset of spermatogenesis, which is blocked by the injection of anti-oestrogen ICI 182,780. On the other hand, when males were treated with a high dose of estradiol or ICI 182,780, disruption of testicular structure, tubular atrophy and increased apoptosis of germ cells were observed [45]. Moreover, an improvement of the recrudescence of spermatogenesis in estradiol treated rodents was observed [46, 47]. The induction of spermatogenesis by estradiol in hypogonadal (hpg) mice involved an ERα dependent neuroendocrine mechanism increasing circulating FSH and premeiotic spermatogonia and meiotic spermatocytes [48]. Neonatal oestrogen administration to rats induced an increase in the number of spermatogonia at day 16 of life [49]. However, experiments conducted with adult rats showed that estradiol effects are dose and age dependent. While estradiol reduced sperm motility even at a low dose, doses below 10 μg/kg/day appeared to maintain whilst higher doses reversibly disrupted spermatogenesis [50]. Chronic estradiol benzoate treated adult rats showed a decrease of pachytene spermatocytes and round spermatids due to high testicular oxidative stress, altered serum hormonal levels (LH, FSH and testosterone) and low intra-testicular testosterone [51]. The exposure of the adult male rat to a high phytoestrogen diet confirmed spermatogenesis disruption by increasing germ cell apoptosis [52] (Fig. 2). These effects on spermatogenesis, apoptosis and hormone levels were maintained in adults after neonatal exposure [53]. Moreover, some works enlightened the possible implication of oestrogens in the regulation of differentiation (spermiogenesis) and thus the quality of the gamete and finally the success of all following steps (epididymal transit, capacitation, acrosomic reaction) until fertilization of the oocyte. In fact, treatment of adult monkeys with an aromatase inhibitor suggests that oestrogens are important for spermatid differentiation [54, 55] (Fig. 2). In rodents, the first sign of effects (deformation of acrosomal granule and nucleus) of neonatal estradiol administration was detected in the steps 2-3 spermatids [56]. Studies conducted in rats by D’Souza group suggest that the elongation process of spermatids from steps 8 to 19 is androgen dependent whereas differentiation of round spermatids from steps 1 to 6 is oestrogen relevant. This can be substantiated by the fact that 20 μg/kg/day of 17β-estradiol administered for 20 days induced prolonged deficiency of testosterone causing an absence of step 9–19 spermatids seen and the lack of apoptosis seen in steps 1–6 round spermatids [57]. In all estradiol treated groups, they observed an elevation of elongated spermatids failure to undergo spermiation demonstrating an inhibiting effect of exogenous 17β-estradiol on spermiation [58] that could be due to altered vimentin phosphorylation and reorganization [59]. However, this group observed that 20 and 100 μg/kg/day of estradiol have a different effect on the number of rat elongating and round spermatids whereas both induced a significant decrease in 2n cells (somatic and germ cells) and 4n cells (pachytene spermatocytes) in accordance with cell apoptosis effect of exogenous estradiol in testis [60]. The observation of abnormal acrosome development in the ArKO mouse suggests that acrosome biogenesis could be an oestrogen dependent process [23] (Fig. 2). This hypothesis is supported by high levels of aromatase in the Golgi complex of the developing spermatid [61] as well as the presence of oestrogen receptors in rat spermatids [5, 15]. The chromatin condensation occurring during spermiogenesis is also regulated by oestrogen (for review [62]). In fact, the levels of testicular and sperm proteins implicated in this step (TP1, TP2, P1 and CREMτ) decreased after estradiol treatment [63]. Cacciola and collaborators [64] have shown that low 17β-estradiol levels in CNR1 knockout mice play an important role in regulating chromatin remodelling of spermatids by interfering with chromatin reorganization. Moreover, oestrogens, by promoting histone displacement and chromatin condensation rescue, were able to efficiently reduce the greater nuclear length observed in Cnr1^-/-^ sperm, a morphological parameter related to chromatin quality [65].

Knowing that effects of estradiol could implicate different oestrogen receptors subtypes, some studies were conducted with some oestrogen receptor subtype specific ligands [66, 67]. The over-activation of ERα or ERβ subtype had detrimental effects on the fertility parameters knowing that the two ERs had overlapping and distinct roles. The activation of ERβ would be notably involved in spermiation process and apoptosis whereas the activation of ERα would be involved in spermiogenesis regulation (Fig. 2).

Pathak and collaborators [68] had observed hypo-methylation at the Igf2-H19 ICR in the spermatozoa of tamoxifen**-**treated rats; this was also found in the methylation patterns of this loci in the embryos (Fig. 2). However, further studies from the same group did not indicate any changes in the methylation status of other imprinted genes (DMR) in spermatozoa [69].

In conclusion*,* in vivo exposures (anti-aromatase, anti-oestrogens or oestrogens) demonstrate that it is probably the balance between oestrogens and androgens which is crucial for the ongoing of spermatogenesis and quality of spermatozoa. In fact, whereas overexposure to estradiol or to SERM like tamoxifen can cause deleterious effects on the male reproductive tract and fertility, estradiol can attenuate the age related decline in spermatogenesis [70, 71].

#### Seasonal breeders

Some natural models of spermatogenesis arrest are the seasonal breeders and in several species the synthesis and somewhat the role of oestrogens have been explored. In the male black bear *(Ursus americanus)*, the presence of aromatase has been reported at the beginning of testicular recrudescence in Sertoli cells and then in round and elongating spermatids in June, in mating season [72]. In Siberian Hamster, oestrogens are able to induce initiation of spermatogenesis, independently of FSH in photo-regressed adult male [47]. In roe deer (*Capreolus capreolus*) a study showed that oestrogens could be implicated in sperm production and in spermatozoa maturation by a regulated expression of ERα [73]. In the wild male ground squirrel (*Citellus dauricus Brandt*) a positive immunoreactivity for aromatase has been evidenced in Leydig and Sertoli cells and all types of spermatogenic cells only during the breeding season and was absent in the non-breeding season. Authors suggest that oestrogens could play an important role in spermatogenesis and testicular recrudescence and regression process [74]. In stallion, although no arrest of spermatogenesis is observed, the semen oestrogens/androgens ratio and ESR expression on spermatozoa in breeding season is higher to the non-breeding season [75, 76].

#### In vitro experiments

The experiences conducted in vitro permitted to precise the events regulated by oestrogens (Fig. 2) and their mechanisms of action. Whereas endogenous oestrogens inhibited male germ cell line development in mice during perinatal life [77], estradiol stimulated the proliferation of rat gonocytes [78, 79]. There is evidence of the direct role of oestrogens in preventing germ cell apoptosis. The protective effects of estradiol observed in cultures of human adult seminiferous tubules develop very quickly, indicating a non-genomic action of oestrogens [80]. However, estradiol was reported to induce in vitro apoptosis of rat spermatogenetic cells via the mitochondrial pathway; these elements tend to demonstrate the direct action of oestrogen in the absence of testicular somatic cells and without the interference of the hypothalamo-hypophyseal axis [81]. More recently, we were able to observe that estradiol activates the EGFR/ERK signalling pathway that modulates the expression of genes involved in the balance between cell proliferation and apoptosis within PS and RS purified by rats’testes [4, 5]. Oestrogens’ effects seem to depend on the cellular environment and estradiol concentration, as we have seen in cultures of adult rat seminiferous tubules, estradiol at 10^-9^ M expresses a different regulation of cyclins A1 and B1 [82]. In cultured immature rat Sertoli cells, 17β-estradiol induces the translocation of oestrogen receptors ESR1 and ESR2 to the cell membrane, as well as MAPK3/1 phosphorylation and their proliferation [83] whereas the binding of estradiol to GPER mediates MAPK3/1 activation through G protein beta gamma subunits that promote SRC-mediated metalloprotease dependent release of EGFR ligands which regulate gene expression involved in apoptosis [19, 84]. The anti-apoptotic effect of 17β-estradiol in immature Sertoli cells was confirmed by Simoes et al. [85]. 17β-estradiol and G1 (agonist of GPER) also induce PI3K/AKT signalling pathway activation and CREB phosphorylation in immature Sertoli cells [84]. A recent publication from the same lab suggests that ESR1 and ESR2 activation by estradiol is respectively involved in proliferation and in Sertoli cells differentiation respectively. In fact, from 15-day-old rats E2 modulates Sertoli cell proliferation through ESR1/NF-kB-mediated increase of CCND1, and cell cycle exit and differentiation through ESR2/CREB-mediated increase of CDKN1B, GATA-1 and DMRT1 [86]. Kumar and collaborators used seminiferous tubule culture to demonstrate that the genes involved in actin remodelling (*Arpc1b, Evl* and *Picalm*), could also play a role in spermiation and are oestrogen-regulated [87].

### Spermatozoa

In addition to spermatogenesis events regulated by oestrogens, these hormones could also regulate some functions of spermatozoa (Table 1). Knowing that spermatozoa are transcriptionally inactive cells [88], the effects of oestrogen are non-genomic. Upon estradiol exposure, Aquila and collaborators observed an enhancement in the phosphorylation of the proteins in the PI3K/Akt pathway, some of them involved in cell survival signals [9]. However, Bennetts and collaborators observed no effect of 17β-estradiol on human spermatozoa viability whereas catechol oestrogens were able to induce a significant loss of cell viability [89]. In the same way, estradiol was described inducing or not DNA damage [89, 90]. 17β-estradiol also stimulated golden hamster spermatozoa motility [91] whereas it reduced the average path velocity (VAP) and the straight-line velocity (VSL) of stallion sperm [92]. In human spermatozoa, only catechol oestrogens (no estradiol) were reported to modify their motility [89] although it was previously said to regulate them positively [93–95]. The activation of ERK1/2 by estradiol could be involved in the positive effects of estradiol [95]. It was recently demonstrated that LRH-1, a transcription factor located in human sperm head, could also be implicated in oestrogen signalling pathway of motility, knowing that oestrogen receptors ESR1 and ESR2 are mainly described on flagellum [9, 11, 14] and without hypothesis of molecular mechanisms [96]. In vivo mice exposure to increased 17β-estradiol concentrations caused premature sperm capacitation in the epididymis [97]. This effect was confirmed by an increase of protein tyrosine phosphorylation level after in vitro incubations of epididymal spermatozoa with 17β-estradiol; but the number of sperm that underwent the acrosome reaction was lower in this group [98].

**Table 1:** Mammalian sperm functions regulated by some selected oestrogens (17β-estradiol, catechol oestrogens, diethylstilbestrol, estrone, estriol, ethynylestradiol, genistein)

Molecule	Event	Species	Effect	Reference
17β-estradiol (E2)	Viability	Human	No effect	[89]
	ROS production	Human	No effect	[89]
	DNA integrity	Human	-/no effect	[89, 90]
	Motility	Human Golden hamster Stallion	+/no effect + -	[14, 89, 91–95]
	Capacitation	Human, mouse, boar bovine	+ No effect	[14, 95, 98–100], [102] ^a^ [103]
	Acrosome reaction	Human Boar Mouse Bovine, stallion	+/no effect + +/- No effect	[92, 95, 98–100], [102] ^a^ [103]
	Progesterone stimulated calcium and induced acrosome reaction	Human	-	[3]
	Fertilizing ability	Mouse	+	[99]
	Lipolytic effect	Human	+	[14]
	Glucose metabolism and insulin secretion	Human	+	[14]
Catechol oestrogens (2OHE2, 4OHE2)	Viability	Human	-/no effect	[89]
	ROS production	Human	+	[89]
	DNA integrity	Human	-/no effect	[89]
	Motility	Human	-	[89]
Diethylstilbestrol (DES)	Viability	Human	No effect	[89]
	ROS production	Human	+	[89]
	DNA integrity	Human	-/No effect	[89, 90]
Estrone (E1,) Estriol (E3), Ethynylestradiol (EE2)	Capacitation	Boar, mouse	+	[98, 101]
	Acrosome reaction	Boar Mouse	+ -	[98, 101]
Genistein	Viability	Human	No effect	[89]
	ROS production	Human	+	[89]
	DNA integrity	Human	-	[90]
	Capacitation	Human, mouse, boar, bovine	+	[99, 100], [102] ^a^ [103]
	Acrosome reaction	Human, mouse, boar Bovine	+ No effect	[99, 100], [102] ^a^ [103]
	Fertilizing ability	Mouse	+	[99]

+: positive effect, -: negative effect and +/-: positive and negative effect

^a^ Effects dependent of the time and the concentration

*ROS* reactive oxygen species

*DNA* deoxyribonucleic acid

Oestrogens and/or xenooestrogens also stimulated in vitro mouse, human, boar and bovine sperm capacitation [95, 98–102], human, mouse, boar and bovine sperm acrosome reaction [95, 99, 102, 103] and the fertilizing ability of mouse sperm suspensions [99]. These effects were reported to be time and species dependent [102] (Table 1) but not involving classical oestrogen receptors [99]. However, it has been observed that oestrogens can inhibit some events induced by progesterone. In fact, estradiol inhibited Ca^2+^ influx and acrosomic reaction induced by progesterone in human spermatozoa (for review [3]) and hyper-activation enhanced by progesterone and melatonin in hamster spermatozoa [104, 105]. In human, 17β-estradiol also regulated cholesterol efflux, protein tyrosine phosphorylation, motility, and acrosin activity of sperm but the impact of E2 treatments were reduced or absent in the case of varicocele [14]. Finally, estradiol was also able to regulate lipid and glucose metabolism of sperm [14].

## 1α,25(OH)_2_Vitamin D_3_ (1,25-D_3_)

### Vitamin D3 receptors

The vitamin D receptor (VDR), originally identified as a chromatin-associated protein [106] binds 1,25-D_3_ with high affinity and specificity, and is associated to 1,25-D_3_ classical effects. On the other hand, the main assumption is that rapid responses to this steroid hormone first occur in the plasma membrane through a membrane-associated receptor [107]. Through crystallography studies, the group of Norman [108–110] showed that besides the genomic binding pocket (VDRnuc), VDR also has an alternative pocket (VDRmem) that could be associated with non-genomic responses initiated in the plasma membrane.

Another membrane-associated receptor for 1,25-D_3_ has also been described. PDIA3 (protein disulfide isomerase family A, member 3) was first isolated from the basal membrane of intestinal epithelial cells of chicks [107]. It was demonstrated that this receptor could bind to 1,25-D_3_ with high affinity, being first nominated 1,25-MARRS (1,25-D_3_ membrane associated rapid response to steroids). This protein also received other denominations, such as ERp60, ERp57 and GRp58. Later on, it was demonstrated that they were one and the same protein, each having an identical structure when compared to others [111]. Like the classical 1,25-D_3_ receptor VDR, PDIA3 is also present in the caveolae at the plasma membrane [112, 113].

Vitamin D and all its metabolites, including the steroid hormone 1,25-D_3_, are conformationly flexible (for review [2]). The conformational flexibility (6-s-*trans* and 6-s-*cis*) determines the signal transduction pathway that has to be activated and, consequently, the biological response produced by its activation (respectively genomic or non-genomic respectively) [109, 114]. Studies about the structure-function of 1,25-D_3_ showed that the 6-s-*cis* analogue vitamin, 1α,25-dihydroxylumisterol_3_ (JN) can efficiently induce transcaltachia in intestinal epithelium and also stimulate Ca^2+^ uptake in osteosarcoma cell line via VDRmem. This analogue, however, failed to activate the genomic action, and it has shown a very low capacity to bind to VDRnuc [115, 116]. On the other hand, another analogue vitamin, the 1β,25-dihydroxyvitamin D_3_ (HL) has been proved to block rapid responses induced by 1,25-D_3_ or JN and is recognized as a specific antagonist of the non-genomic action [117, 118]. Similarly, the 1,25-D_3_ genomic response can be blocked by co-incubation with the analogue vitamin (23S)-25-dehydro-1α(OH)-vitamin D_3_-26,23-lactone (MK), which antagonistic action is caused by the inhibition of heterodimer formation between VDR and RXR, and of VDR interaction with co-activator, steroid receptor co-activator 1 (SRC-1) [119].

The presence of VDR in several tissues and cells of the male reproductive system has been demonstrated by several studies; this could explain the importance of this hormone in reproductive tissues. Animal studies have shown VDR expression in both nucleus and cytoplasm in primary cultures of immature rat Sertoli cells and in immature mice Sertoli cell line TM4 [120–122], seminiferous tubules and the caput epididymis [123, 124]. A staining of Leydig cells was also observed for human and mouse [125, 126]. VDR is expressed in germ cells of both rodents and humans: spermatogonia, spermatocytes and round and elongated spermatids [121, 125, 126] as well as in sperm [127, 128] but Sertoli cells are still considered to be the main 1,25-D_3_ target in the adult testis [2] (Fig. 1a). Previous studies have shown that, after injection of [3H]-1,25(OH)_2_-vitamin D_3_ (soltriol), the nuclear labelling is found in Sertoli cells, and is the highest one proportionally to the seminiferous tubules in which residual bodies in the apical part are distinctly stained [129]. ERp57, before being described as a vitamin D receptor, was investigated in human testis and spermatozoa. ERp57 was located in spermatocytes to spermatozoa and Leydig cells; a faint labelling was also observed in Sertoli cells [130] (Fig. 1). In the rat testis, PDIA3 is also present in Leydig and germ cells (from spermatogonia to spermatozoa). PDIA3 protein was observed on the entire membrane of rat spermatozoa and in the acrosome [131].

Spermatozoa are highly compartmentalized cells, and all studies investigating VDR expression in mature human spermatozoa showed VDR expression in the post-acrosomal part of the head, mid-piece, and in the neck region (Fig. 1b) [125, 127, 128]. Subcellular VDR expression may therefore depend on optimal spermatogenesis and maturation, because incomplete formation of the subcellular compartments may lead to an unusual localisation and, potentially, an unusual function of the receptor [132, 133].

### Roles, effects and signalling pathways of 1,25-D_3_ in testis and spermatozoa

#### KO mice models and in vivo treatments

There is accumulating evidence that 1,25-D_3_ and VDR constitute an important part of the reproductive tissues. In fact, although the male vitamin D receptor of null mutant mice produced by Erben and collaborators were fertile [134]; the VDR knockout mice produced by the group of Kinuta [135] showed gonadal insufficiencies. Indeed, in the male, decreased sperm count and decreased motility with histological abnormality of the testis were observed [135]. This difference may be related to a different genetic background [134]. Early studies in vitamin D-deficient male rats have shown that, even if they are able to reproduce themselves, animals have a 45% reduction in successful mattings as well as a decreased overall fertility rate that is reduced by 73% when compared to controls [136]. The testes of vitamin D-deficient rats showed incomplete spermatogenesis and degenerative changes [137]. The fertility of vitamin D deficient male rats is restored by treatment with vitamin D and its active metabolite 1,25-dihydroxycholecalciferol but their effect is indirect as fertility is also restored by a diet supplemented with high levels of calcium [138]. 1,25-D_3_ also had some protective actions in testis of diabetic rats. Hamden and collaborators [139] have observed that the administration of 1,25-D_3_ 3 weeks before and after diabetes induction prevented oxidative stress, toxicity and hypo-fertility in diabetic rat testes. Finally, it has been shown that vitamin D treatment up-regulates some testis-specific genes in cryptorchid mouse testis whereby 19 out of 2483 testis-specific genes showed upregulation by 1,25-D_3_ treatment [124].

#### In vitro treatments

As observed in osteoblasts notably by Enjuanes et al. [140], we observed that 1,25-D_3_ is able to stimulate the expression of *Cyp19*, the gene which encodes the enzyme P450 aromatase, in immature rat Sertoli cells [121]. Menagaz et al. [141] reported that 1,25-D_3_ plays an important role in testis through genomic effects that can be triggered by protein kinase A, as well as by rapid responses (amino acid accumulation) involving Ca^2+^/K^+^ channels on the plasma membrane and stimulating exocytosis via Cl^−^channel activation in the Sertoli cell line TM4 [120]. Other studies demonstrated that 1,25-D_3_ triggers plasma membrane-initiated actions by modulating calcium uptake and by altering gamma-glutamyltranspeptidase (GGTP) activity in immature rat testis. GGTP is involved in the synthesis of specific proteins known to be secreted by Sertoli cells [142]. Also, Rosso et al. [143] reported that 1,25-D_3_ activates p38 MAPK and reorganizes microtubules, involving Ca^2+^, PKC and ERK1/2 as upstream regulators, and that extracellular Ca^2+^ have a central role to rapidly start hormone-induced gene transcription and/or the secretory activity of Sertoli cells.

#### Spermatozoa

In a study investigating human sperm at the molecular level, 1,25-D_3_ had an effect on cholesterol efflux, protein phosphorylation, and increased sperm survival [128]. Thus, 1,25-D_3_ might play an important role in the extra testicular maturation of sperm by influencing capacitation and might modulate sperm survival. Also, Aquila et al. [144] demonstrated that 1,25-D_3_ through VDR increased intracellular Ca^2+^ levels, motility, and acrosin activity, and reduced triglyceride content in sperm, revealing an effect of 1,25-D_3_ in the acquisition of fertilizing ability in human sperm. Moreover, 1,25-D_3_ increased sperm motility and induced acrosome reaction [145]. 1,25-D_3_ effect on motility was dependent on the characteristics of samples as 1,25-D_3_ significantly increased spermatozoa motility in young men but was unable to do so in spermatozoa from sub-fertile men [133].

## Interactions between oestrogens and 1,25-D_3_ signalling pathways

**Fig. 3:**
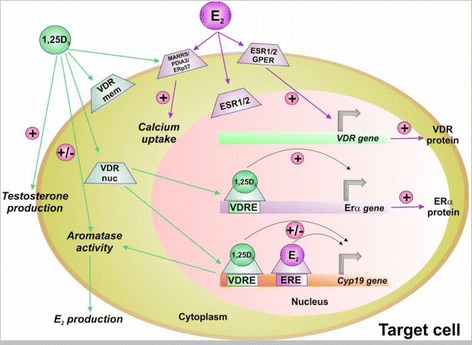
Interactions between genomic and nongenomic action of 1,25-D_3_ and oestrogens.1,25-D_3_ can bind VDR localized at the plasma membrane or intracellular VDR and could regulate *Cyp19* and *ERα* gene expression, aromatase activity and estradiol production. Estradiol can regulate VDR gene expression by ESR2 localized at the plasma membrane and *Cyp19* gene expression. E2: estradiol, 1,25-D_3_: 1α,25 dihydroxyvitamin D_3_, ESR1/2: estrogen receptor 1 and 2, GPER: G protein coupled estrogen receptor, VDRmem: vitamin D receptor localized at the plasma membrane, VDRnuc: nuclear vitamin D receptor, VDRE: vitamin D responsive element, ERE: oestrogen responsive element

As reported before, oestrogens and 1,25-D_3_ could regulate spermatogenesis or spermatozoa’s maturation, suggesting possible interactions between their signalling pathways. This idea is, in fact, supported by some already published data (Fig. 3). First, both hormones’ respective receptors could be expressed by the same cells (testicular cells and spermatozoa) (Fig. 1). Then, VDR ablation was responsible for oestrogen deficiency and abnormal spermatogenesis observed in VDRKO mice whereas oestrogen supplementation protected the testis of histological abnormality [135]. Moreover, a significantly lower ERβ expression was found in testis of Vdr^+/-^ and Vdr^-/-^ Leuven strain mice without changes in the histology of the testis whereas epididymal expression of ERα and the oestrogen target gene *Aqp9* were higher [146]. 1,25-D_3_ reduced significantly the levels of the steroid receptors (ERα, PRA and PRB) in human uterine leiomyoma (HuLM) cells, regulated positively the levels of its receptor (VDR) and inhibited oestrogen induced proliferation of cultured (HuLM) cells suggesting that 1,25-D_3_ could function as anti-oestrogenic/progesteronic agent [147].

1,25-D_3_ could also regulate seric steroid levels and notably estradiol levels. In fact, 1,25-D_3_ positively regulated *Cyp19* expression in immature rat Sertoli cells [121] as previously observed in human osteoblasts [140, 148]. Estradiol production of rat costochondral chondrocytes [149] and aromatase activity of human mesenchymal stem cells [150] and prostate cancer cells [151] are stimulated by vitamin D. However, 1,25-D_3_ effect on oestrogen metabolism is tissue specific. Admittedly it had a negative effect on aromatase expression and estradiol production in MCF-7 cells, whereas it had no effect on estradiol production in LnCap and NCI-H295R. Nevertheless, it induced a significant increase of estradiol production in NCI-H295R in presence of additional androstenedione [152]. 1,25-D_3_ also had a positive effect in estradiol production in osteosarcoma and ovarian cancer cells, but resulted a negative effect in oestrogen receptor-positive breast cancer (BCa) cells and adipocytes [153]. Aromatase down regulation by 1,25-D_3_ in BCa cells was due to a direct repression of aromatase transcription via promoter II through the vitamin D-response elements identified in this promoter and an indirect suppression by reducing the levels of prostaglandins [153]. It is interesting to note that *Cyp19* expression in male gonad is in part driven by the promoter PII (for review [154]). The analogue vitamin D EB1089 is also able to decrease *Cyp19* gene expression and aromatase activity and to inhibit the aromatase dependent cell growth of breast cancer cells [155]. In rat granulosa cells, 1,25-D_3_ reduced testosterone induced aromatase expression but improved 17β-estradiol production by a calcium dependent pathway [156]. In human activated macrophages, 1,25-D_3_ may downregulate the pro-inflammatory cytokine production by significantly decreasing the aromatase activity, especially in presence of an estrogenic milieu [157]. In HEK-293, ERK1/2 phosphorylation and up regulation of VDR protein expression induced by estradiol required the association of ERβ to plasma membrane caveolae components [158]. 1,25-D_3_ is able to restore the expression of a functional ERα in ER negative breast cancer cells probably by a transcriptional VDR dependent regulation, as the ER promoter contains several putative vitamin D response elements [159]. 1,25-D_3_ also regulates androgen production, the aromatase substrate, in a tissue specific manner [152, 160, 161]. It was interesting to note a testosterone level increase in a group of men who received vitamin D supplementation [160]. 1,25-D_3_ had a protective effect on alloxan-induced damage in reproductive system by enhancing the testosterone and 17β-estradiol levels consequently protecting from oxidative stress, cellular toxicity and maintaining the number and motility of spermatozoids [139]. Matsuda and collaborators have also demonstrated that the effects of oestrogen on mouse vaginal development are influenced by 1,25-D_3_ [162]. Finally, it was observed that in a reducing environment, estradiol competed for binding to 1,25-D3-MARRS receptor/PDIA3/ERp57 and was able to stimulated calcium uptake in isolated enterocytes [163].

Therefore, in testicular cells and in spermatozoa, vitamin D could modulate oestrogen non-genomic effects but also their genomic effects and reciprocally.

## Conclusions

To conclude, we assume that common testicular cells and spermatozoa express oestrogen receptors (ESR) and 1,25-D_3_ receptors (VDR); moreover**,** in vitro and in vivo studies suggest that oestrogens and 1,25-D_3_ play roles in spermatogenesis and spermatozoa functions**.** However, the mechanisms involved in the regulation of the events under 1,25-D_3_ and 17β-estradiol control; and their possible interactions could not be completely identified. Indeed, even if the recent review published by Blomberg Jensen [164] also indicated a relation between VDR and oestrogen in the male reproductive organs, there were few data about dialogue mechanisms between oestrogen and vitamin D signalling pathways. Some data obtained in numerous cell types suggest that calcium could play an intermediate role in non-genomic effects, notably in spermatozoa but also in other testicular cell types like germ cells and Leydig cells. In fact, these cells**,** in which calcium currents were measured express VDR and ESR [165, 166]. The physiological significance and the specific roles endorsed by these hormones and their receptors in human spermatozoa require further investigation. A better comprehension of the matter could give us information regarding the potential altering effects of environmental xenobiotics (and notably xenoestrogens) on male fertility [167]**.** It has been stated that serum 1,25-D_3_ at high (>50 ng ml^-1^) and low (<20 ng ml^-1^) levels can be negatively associated with semen parameters [168] and that seminal plasma estradiol levels have shown an increase in infertile men [169]; but without identifying any pathophysiologic features in relation with male fertility**,** the interest to carry out such studies in men becomes all the more relevant**.** Moreover, in 2012, Lerchbaum and Obermayer-Pietsch [170] as well as Boisen and collaborators in 2016 [171] indicated that other studies (notably high quality randomized controlled trials) would be necessary to evaluate the effect of vitamin D supplementation on the improvement of semen quality and subsequently on fertility.

## Abbreviations

1,25-D_3_: 1α,25(OH)_2_-vitamin D_3_
E_2_: Estradiol
ESR: Estrogen receptor
GPER: G protein-coupled estrogen receptor
PS: Pachytene spermatocyte
RS: Round spermatid
VDR: Vitamin D receptor
VDRmem: VDR localized at the plasma membrane
VDRnuc: VDR localized in the nucleus
